# Societies to genes: can we get there from here?

**DOI:** 10.1093/genetics/iyab104

**Published:** 2021-07-29

**Authors:** Robert E Page

**Affiliations:** 1 School of Life Sciences, Arizona State University, Tempe, AZ 85287-4501, USA; 2 Department of Entomology and Nematology, University of California, Davis, Davis, CA 95616, USA

**Keywords:** genotype–phenotype architecture, honey bee social behavior, behavior genetics, forward genetics, pollen hoarding, quantitative trait locus, pleiotropy, epistasis, epistatic pleiotropy

## Abstract

Understanding the organization and evolution of social complexity is a major task because it requires building an understanding of mechanisms operating at different levels of biological organization from genes to social interactions. I discuss here, a unique forward genetic approach spanning more than 30 years beginning with human-assisted colony-level selection for a single social trait, the amount of pollen honey bees (*Apis mellifera* L.) store. The goal was to understand a complex social trait from the social phenotype to genes responsible for observed trait variation. The approach combined the results of colony-level selection with detailed studies of individual behavior and physiology resulting in a mapped, integrated phenotypic architecture composed of correlative relationships between traits spanning anatomy, physiology, sensory response systems, and individual behavior that affect individual foraging decisions. Colony-level selection reverse engineered the architecture of an integrated phenotype of individuals resulting in changes in the social trait. Quantitative trait locus (QTL) studies combined with an exceptionally high recombination rate (60 kb/cM), and a phenotypic map, provided a genotype–phenotype map of high complexity demonstrating broad QTL pleiotropy, epistasis, and epistatic pleiotropy suggesting that gene pleiotropy or tight linkage of genes within QTL integrated the phenotype. Gene expression and knockdown of identified positional candidates revealed genes affecting foraging behavior and confirmed one pleiotropic gene, a tyramine receptor, as a target for colony-level selection that was under selection in two different tissues in two different life stages. The approach presented here has resulted in a comprehensive understanding of the structure and evolution of honey bee social organization.

## Introduction

The goal of behavioral genetics is to understand *why INDIVIDUAL A does thing X while INDIVIDUAL B does thing Y*. What are the genetic factors involved and what are the relative contributions of the genotype, the environment, and their interactions. The goal of social behavioral genetics is to explain *why GROUP A does social thing X while GROUP B does social thing Y.* Social behavioral genetics is more complex because not only are the genotypes of the individual social members' important factors in determining their behavioral phenotype but also their complex interactions with social partners with different, though usually related, genotypes that create and affect their own social environments (Linksvayer and Wade 2005; Linksvayer *et al.* 2009; Linksvayer 2015). The genetics of social behavior looks at social phenotypic traits that are emergent—not expressed in single individuals.

Studies of the genetics of complex social behavior have been mostly restricted to the social insects. Approaches have varied (Rittschof and Robinson 2016). Some use a reverse genetics approach where a gene or genes of known function in solitary species, such as *Drosophila melanogaster*, are knocked down in individuals and effects on individual and social behavior are sought (Ben-Shahar *et al.* 2003; Ament *et al.* 2012). Forward genetic approaches, behavior to the gene, come in two “flavors.” The most commonly used method begins with gene expression studies between two groups that vary in some behavior that is involved in colony social structure. Discovered genes are knocked down and effects on individual behavior are studied (Wang *et al.* 2009, 2020; Page *et al.* 2012). The other forward genetics method takes advantage of human-assisted selection and quantitative trait locus (QTL) mapping. This method draws upon a broad base of experimental methods from behavioral science, transmission genetics and breeding, physiology, quantitative and molecular genetics, and complex systems “thinking,” and leads to a comprehensive understanding of social behavior and genetics.

I studied the behavioral genetics of pollen storage in honey bees for more than 30 years (Page *et al.* 2012). The effort was collective with my technician and colleague Kim Fondrk and numerous students and postdoctoral researchers. We used a forward genetic approach and employed bidirectional, human-assisted selection that resulted in the establishment of two strains that varied in their expression of a social phenotype. We focused on just one trait, the amount of pollen stored in the nest, a social trait that is a consequence of the interactions of thousands of bees. There are about 10–40 thousand worker bees in a honey bee colony, depending on the time of year. Honey bee colonies have a reproductive division of labor where the single queen normally lays the eggs while the workers are facultatively sterile. They also have a division of labor among the workers. Roughly half of them are foragers, a behavior that begins when an individual is about 2–3 weeks old. Individual foragers primarily collect pollen (protein) and nectar (carbohydrate). They specialize by biasing their foraging behavior for one or the other. Specialization is partly determined by their genotype. Roughly 1/3rd of the foragers collect pollen, depending on resource availability and time of year. Pollen is collected from flowers and stored in cells of wax comb near the “nursery,” the central part of the nest where eggs are laid in cells and larvae develop into adults. A full-sized colony may contain 10,000 larvae. Pollen is stored in excess of immediate need, it is hoarded. Some bees in the nest consume the pollen in the cells, convert the pollen proteins into vitellogenin (VG) and other proteins in the fat body that then enter the hemolymph, travel to the hypopharyngeal glands, and are converted to glandular secretions that are fed to larvae. Young larvae produce pheromones (chemical signals) that stimulate some of the foragers to collect pollen (Traynor *et al.* 2015). The amount of pollen stored, the number of cells that are full, affects the number of larvae that are raised and inhibits pollen foraging, thus stored pollen is regulated (Fewell and Winston 1992). Returning pollen foragers perform recruitment dances that communicate the distance and direction from the nest to their pollen sources. Other bees attend the dances and are recruited. So, the amount of stored pollen is dependent on the interactions of thousands of individual adults and larvae and is not a phenotypic trait of any individual—it is a social phenotype.

For each of 42 generations of bidirectional selection, one or two per year, we measured colony- and individual-level phenotypic traits that are associated with stored pollen (Page *et al.* 2012). However, selection of the parents each generation was dependent only on the pollen measurements. This allowed us to independently study the phenotypic and genetic architectures of the trait, and the changes that took place at the different levels of biological organization—gene, physiology, anatomy, behavior, and social structure. Collectively they are the elements that make up the pollen-hoarding trait. Traits that demonstrated differences between the strains were independently verified by studies of “wild-type” bees—bees not from either strain—to ensure that differences between strains were not consequences of genetic drift when the strains were selected from the foundation population, or from strong selection. QTL maps were constructed for colony and individual level traits that varied between the strains. Individual QTL maps were compared within and across mapped traits. Confidence limits were estimated for map locations of significant QTL and the DNA sequences contained within were searched for predicted genes (Weinstock *et al.* 2006; Hunt *et al.* 2007). Our goal was to determine which genes or genetic elements contained in the QTL were targets of our selection. Gene lists were made for all significant, annotated genes, and candidates were qualified and assigned based on reason. Association and gene expression studies coupled with gene knockdowns were performed with candidate genes to further qualify the list.

## Phenotypic architecture

### Colony level traits

**Figure 1:**
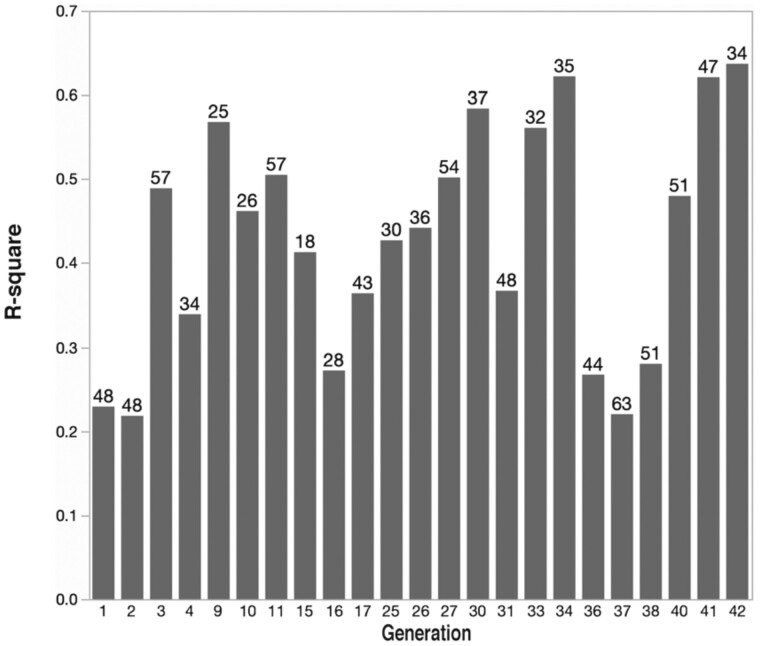
*R* ^2^ values for the pollen hoarding selection program over 42 generations. Values represent the proportion of the total variance in the population, high and low strains combined, that is a result of belonging to different strains. Numbers at the top of bars are the number of total colonies tested. Not all generations are shown as a result of special mating designs used for different research questions such as: a hybrid cross in generation 5; inbreeding in generations 12–14, 18–21, and 24. In addition, no records exist for generations 6, 7, 8, 22, 23, and 35. Outcrosses were made to the source population in generations 6, 8, 15, 22, 27, 35, and 39. All *R*^2^ values are statistically significant with probabilities ranging between 0.0045 and <0.0001.

The initial selection took place in a single commercial population (Hunt *et al.* 1995). High and low subpopulations, designated high and low pollen-hoarding strains, were established and subsequently maintained as closed breeding populations except for occasional outcrosses to the original founding population. The response to bi-directional selection was significant after just one generation. By generation 3, high strain colonies on average contained 6 times more pollen, 21 times more in generation 30. The proportion of the total explained variance in the sample of all colonies is a better measure of selection response. It is similar to heritability, but for entire colonies, and in the strict sense violates the assumptions of the individual heritability models. It provides a measure of the proportion of the variance that is a consequence of belonging to different strains (Figure 1). Because the two strains were selected from the same population, it also is a measure of the selectability of the trait.

### Individual level traits

More than 50 published, independent studies of individual traits were used to map the phenotypic architecture of pollen hoarding (Page *et al.* 2012). Most of these studies were derived from what we called common garden experiments where individuals from different sources, pollen hoarding strains or others, were raised together in common “neutral” colonies. Many trait differences were found to be associated with each other in a complex web revealing an integrated phenotype that is responsible for the social trait.

### Phenotypic map

**Figure 2:**
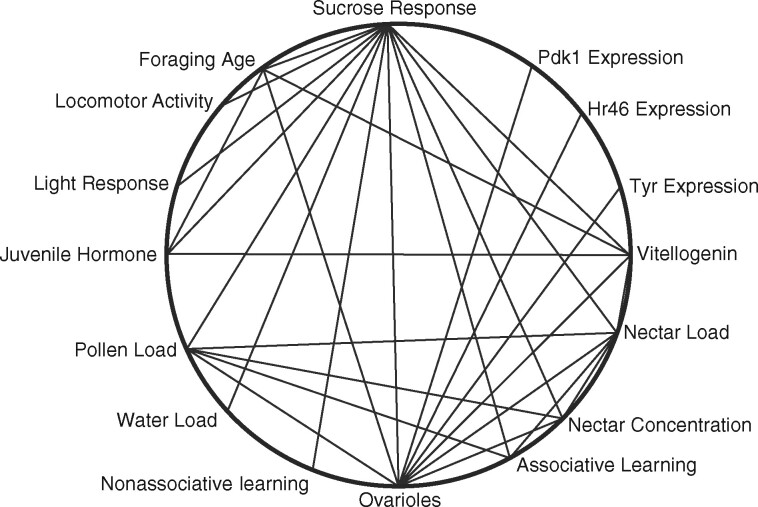
The phenotypic architecture (correlative structure) of pollen-hoarding. Phenotypic traits span levels of biological organization from the genotype to foraging behavior. Lines connect traits that have been demonstrated to be significantly correlated. Studies were performed on high and low-strain workers as well as wild-type bees. All traits shown here vary between bees of the high and low-pollen-hoarding strains. What is most interesting are the connections of vitellogenin, an egg yolk and reproductive signaling protein, with the ovary function, and the response of bees to sucrose. Sucrose response is just an easily obtained measure of the sensory sensitivity of a bee and correlates with many other phenotypic traits. ( From Page (2013), Figure 5.14 .

A phenotypic map of the correlative structure of traits was assembled from a combination of studies of the selected strains as well as wild-type bees from different populations. All traits are shown in the map (Figure 2) varied between the high and low strains and between wild-type pollen and nectar foragers. Therefore, the integration of the phenotypic architecture cannot be explained by linkage disequilibrium resulting from selection, or genetic drift. The map reveals an integrated network of traits associated with foraging behavior and sensory and reproductive physiology with critical nodes of ovariole number and sucrose responsiveness. Bees that are more likely to collect more pollen are more likely to have ovaries composed of more ovarioles and to be more sensitive to sugar.

VG and juvenile hormone (JH) are involved in reproductive signaling and regulate the onset of foraging behavior. When VG titers fall and JH titers rise, a bee initiates foraging (Amdam and Omholt 2003). High VG causes bees to be more responsive to sugar and primes them to bias their foraging for pollen (Nelson *et al.* 2007, Figure 3). Bees with more ovarioles produce more VG, forage earlier in life, and bias their foraging for protein (pollen), thus showing a link between reproductive anatomy and physiology, and foraging behavior. We used dsRNA and knocked down VG in young adult workers. The knockdown phenotypes foraged earlier in life, were more responsive to low concentrations of sugar solution, and were more likely to forage for nectar.

**Figure 3:**
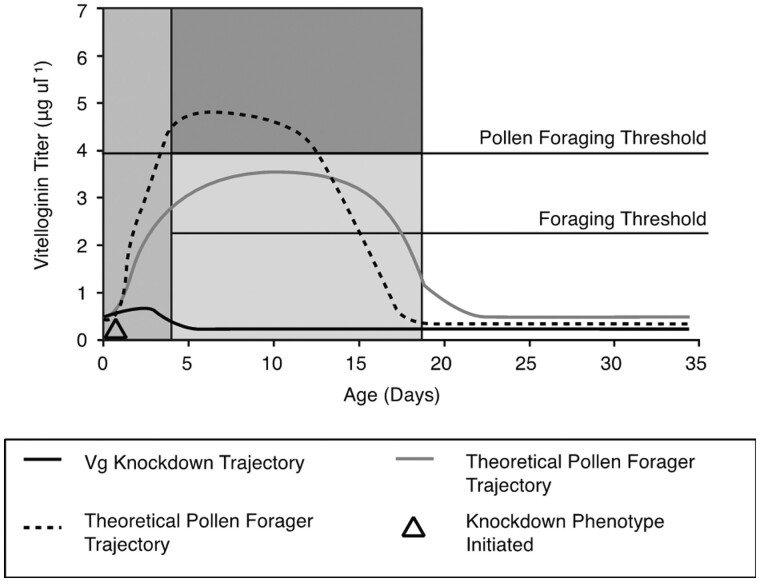
A model for the action of VG in foraging division of labor. Very young bees are unable to forage. They must pass through an initial maturation phase during which flight muscles develop and the cuticle hardens. During this phase (gray) workers are primed for their future foraging specialization by titers of the protein vitellogenin (VG). VG titers above the pollen threshold prime workers for pollen foraging (dark gray), while workers with lower preforaging titers (light gray) are primed for nectar foraging. In workers, VG suppresses the transition from nest tasks to foraging activity when its titer remains above the foraging threshold level. Below this threshold, the probability of initiating foraging is increased. RNAi mediated knockdown of vitellogenin results in workers that mature with vitellogenin titers that are below both the pollen and foraging thresholds, resulting in bees that are more responsive to sugar, forage precociously, and preferentially collect nectar. From Nelson *et al.* (2007), Figure 5.

VG is produced in the fat body of bees. It is an egg yolk protein that is released into the blood, the hemolymph, circulates, and is incorporated into ovaries that have been activated for reproduction. Ovaries produce ecdysteroids, hormones, that initiate VG production in the fat body (Amdam *et al.* 2010). Workers normally have functionally inactive ovaries, but under certain conditions, such as the absence of a queen and larvae in a colony, they develop active ovaries, produce VG, and can make and lay eggs. We proposed that pollen and nectar foraging, and by extension pollen hoarding behavior, is regulated by ancient reproductive signaling networks still operating in worker honey bees that originated before the rise of the insects (Amdam *et al.* 2004, 2006). Change in behavior with changes in reproductive state is ubiquitous, found throughout the Animal Kingdom, including us. A common pattern in insects is to forage for carbohydrates for sustenance when not reproductive, and for protein when reproductively activated (Amdam *et al.* 2004). Our phenotypic architecture suggested a key role of the ovary and VG in tuning the sensory system of workers and directing foraging behavior. When we overlaid the genetic architecture (see below) on the phenotypic architecture we saw why.

### Genetic determination of phenotypic traits

**Figure 4:**
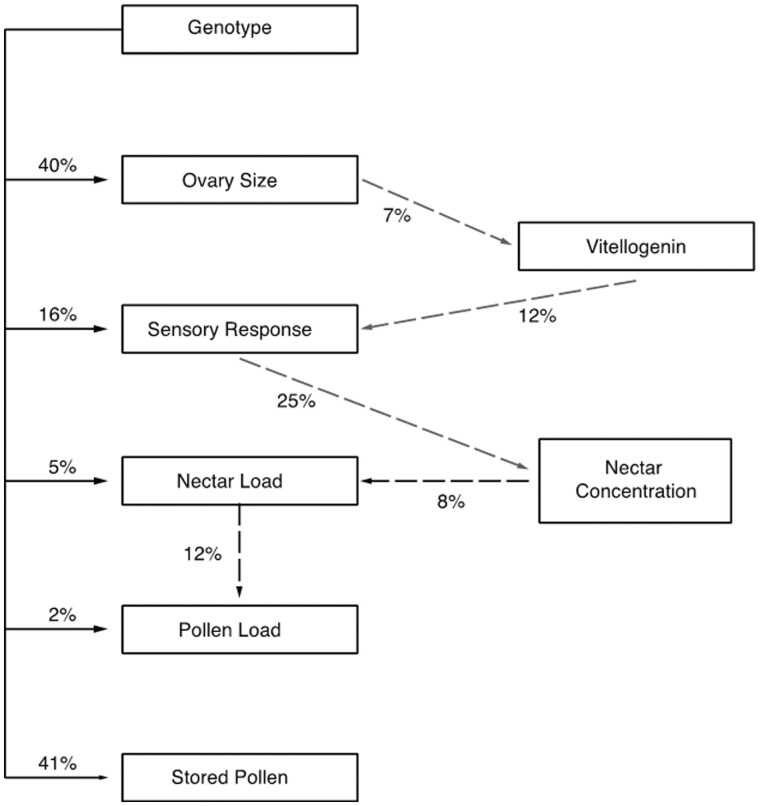
Degree of determination of effects of components of the phenotype architecture of pollen hoarding depicted as stored pollen in the bottom box. Genotype effects, with genotypes specified as high or low strain bees, are shown with solid lines down the left margin for each of the traits on the left. Arrows show the directionality of the effects. Dashed lines show the amount of variance in one trait explained by another. Dark dashed lines were measured in high and low-strains and wild-type bees, light dashed lines were measured in wild-type bees only. From Amdam *et al.* (2009), Figure 6.

We mapped genetic and environmental determinants of a subset of phenotypic traits (Amdam *et al.* 2009). Genotype (high or low strain) explained about 41% of the variance in stored pollen, a social trait. It explained 2 and 5% of the variance, respectively, for pollen and nectar loads—individual traits expressed in a social environment (Figure 4). This was opposite to our initial expectation that individual behavior is “closer to the genes” and would be more selectable than a complex social trait. Stored pollen is regulated. Because it is regulated, it provides a more repeatable measurement of the phenotype with less unexplained variance, hence a higher degree of genetic determination. Individual behavior is performed in a highly variable environment that is determined by the temporal and seasonal availability of nectar and pollen resources, competition for resources, chance discovery of resources to exploit, and experience. Selectability is low. The anatomical trait ovary size (number of ovarioles) is likewise regulated by developmental processes and yields a degree of genetic determination equal to the social trait. We would not have been successful if we had tried to select for pollen hoarding by directly selecting on the individual behavioral traits. In a sense, we reverse engineered the phenotypic and genetic architectures of individuals by selecting on a colony trait.

## Genetic architecture

### Mapping social and individual traits

We constructed genotype–phenotype maps using QTL (Wagner and Zhang 2011). QTL maps of pollen hoarding and individual behavioral, anatomical, and physiological traits revealed a complex genetic architecture. The first map was of the social phenotype, pollen hoarding. We detected two significant QTL, *pln1* and *pln2*, responsible for an estimated 55% of the variance in the amount of pollen stored in the colony (Hunt *et al.* 1995), though there are inherent upward biases in these estimates (Beavis 1994). Two more maps of pollen hoarding, on two populations of bees revealed two additional QTL, *pln3* and *pln4*. We checked for the QTL effects on individual foraging behavior and found significant foraging effects associated with all mapped QTL suggesting that the selected pollen hoarding effects were at least in part due to changes in the foraging behavior of individual workers, as expected. QTL maps were constructed for individual behavior and for anatomical and physiological traits (Table 1).

**Table 1:** QTL maps

Map	Year	Map level	Cross	Number of colonies or individuals	Traits mapped	Marker type	Number of markers	Map size
1	1995	Colony	HBC	38	Pollen hoarding	RAPD	364	3100 cM
2	2000	Colony	HBC	179	Pollen hoarding	RAPD + STS	NR	NR
3	2000	Colony	(A × E) × E*a*	153	Pollen hoarding	RAPD + STS	NR	NR
4	2004	Individual	HBC	182	Foraging behavior	AFLP + STS	387	3900 cM
5	2004	Individual	LBC	94	Foraging behavior	AFLP + STS	396	3700 cM
6	2006	Individual	HBC	96 of 359*b*	Sucrose response	AFLP + STS	405	5376 cM
7	2006	Individual	LBC	95 of 354*b*	Sucrose response	AFLP + STS	471	5141 cM
8	2006	Haploid drones	hybrid	191 of 1,007*b*	Sucrose response	AFLP + STS	417	5310 cM
9	2009	Individual	(A × E) × A	24(3 pools of 8)*c*	Ovarioles	SNP	486/257^*e*^	
10	2009	Individual	(A × E) × A	24(3 pools of 8)*c*	Ovarioles	SNP	486/257^*e*^	
11	2009	Individual	(A × E) × A	48*d* (combined pools of 9 and 10)	Ovarioles	SNP	486/257^*e*^	
12	2011	Individual	HBC	160	Ovarioles	SNP + micro-satellites	231	About 3,873 cM
13	2011	Individual	LBC	160	Ovarioles	SNP + micro-satellites	221	About 3,936 cM
14	2015	Individual	HBC	189	Ovarioles and VG/JH inter-action	SNP	1,125	5,696 cM

a A, Africanized honey bee; E, European honey bee.

b Selective genotyping was used. Only individuals with the more extreme phenotypes were genotyped.

c Individuals with extreme high and low phenotypes were pooled into high and low pools. The pools were genotyped.

d Combined pools from 9 and 10

NR, not reported.

Citations for maps: Hunt *et al.* (1995); Page *et al.* (2000); Rueppell *et al.* (2004a, 2004b, 2006, 2011); Rueppell (2009); Linksvayer *et al.* (2009); Graham *et al.* (2011); Wang *et al.* (2009); Ihle *et al.* (2015).

**Figure 5:**
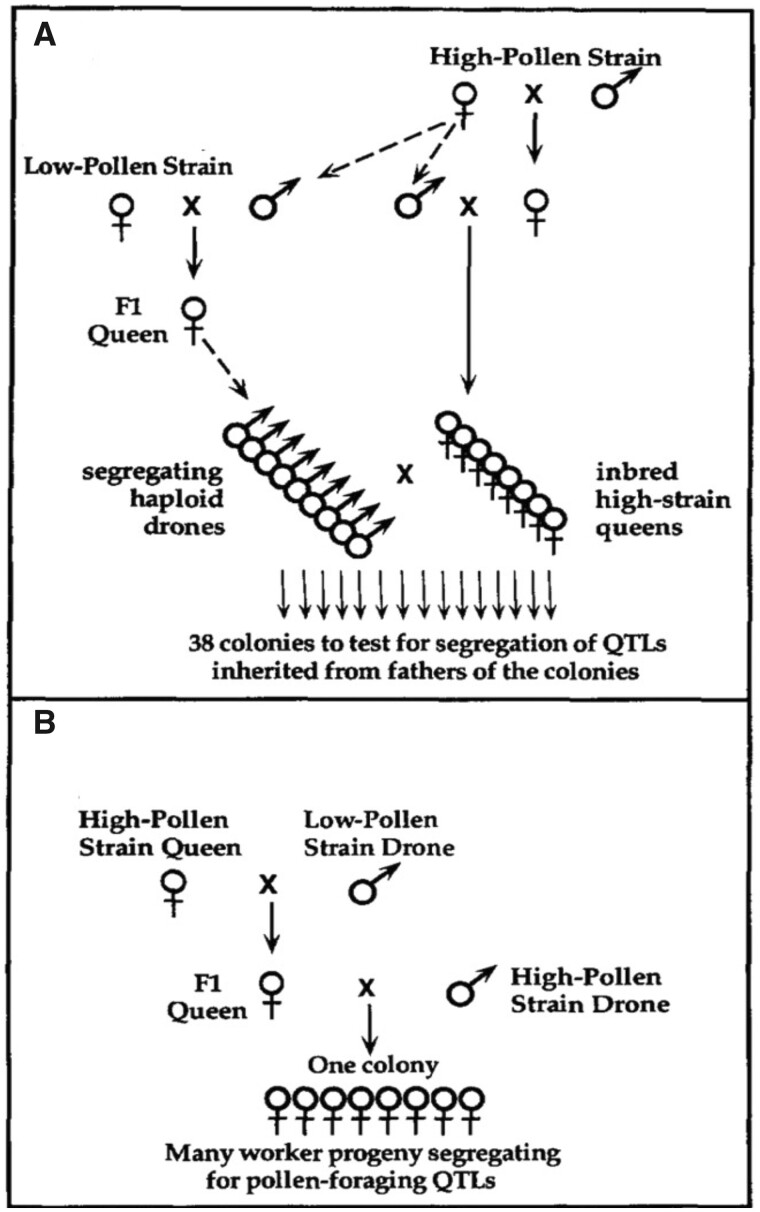
Backcross schemes for studying pollen-foraging quantitative trait loci (A) Crossing scheme for detection and mapping of colony-level QTL for pollen-foraging (measured as area of pollen stored in combs) was based on the contribution of QTL from segregating haploid drone fathers of each colony. The cross was made to produce an FI queen. Segregating haploid drones were each individually mated to a single virgin queen. The source of unfertilized eggs resulting in haploid drones is shown by dashed arrows. (B) Crossing scheme to confirm effects of QTL on individual foraging behavior based on segregation in worker progeny of the Fl queen in a single colony (From Hunt *et al.* 1995, Figure 1).

The haplodiploid sex determination of honey bees aided mapping because males contain a single genome derived from their queen mother. Therefore, colonies derived from single-drone instrumentally inseminated queens had reduced genetic variance within them. The drones were “brothers” derived from the same queen, representing meiosis within the drone mother. Queens were sisters from a single, inbred queen, reducing variance between colonies that was derived from the queen mothers. We assigned the colony phenotypes to the drones for mapping (Figure 5). We were initially hindered from completing more saturated maps by the limitation of available markers, but that improved over time with the development of better marker systems. We were also limited in the number of meiosis we could map for our colony traits because a phenotype was an entire colony, each of which requires much care, perhaps like maintaining cattle. That also improved in time as we found individual behavior traits to serve as surrogates for the colony trait.

**Figure 6:**
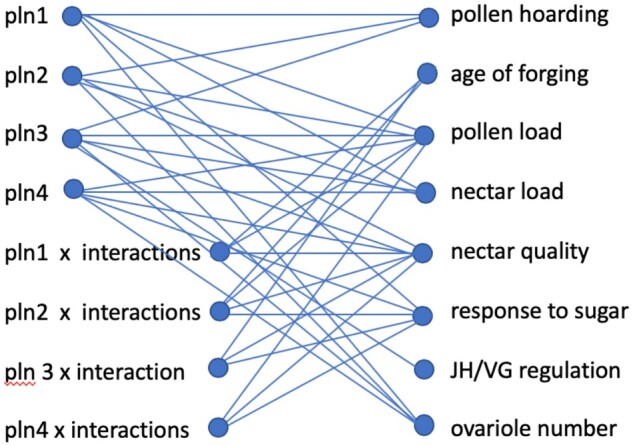
Genetic architecture of effects of *pln1-4* on phenotypic traits. Connecting lines are statistically significant correlations. Chromosome locations for pollen QTL: *pln1* (13:3.5), *pln2* (1:16.5), *pln3* (1:9.2), and *pln4* (13:9.0).

Broad pleiotropy and epistasis were demonstrated directly by the mapping (Figure 6). QTL tends to overestimate pleiotropy because different linked genes contained in them may have effects on the different phenotypes. Genes may also become assembled into linkage groups within QTL by selection to improve their joint effects on a phenotype or phenotypes, supporting functional modularity of phenotypic traits (Murren 2012). Genetic pleiotropy is expected to be restricted to phenotypic traits that function together (Wagner and Zhang 2011), as we found. Our mapped QTL contained relatively few genes, a total of 113 identified when we compared against the draft genome sequence and 219 when compared with the latest assembly OGS3.2, reducing the chance that the observed QTL pleiotropy resulted from the chance linkage. In addition, we constructed 14 QTL maps (Table 1) over many generations including outcrossed populations and populations that were unrelated, many opportunities to break up linkage and re-assort genes, and got consistent results with respect to the location of the QTL (Page *et al.* 2012).

We found other QTL for the individual level traits but our interest remained focused on those that were initially mapped for pollen hoarding and foraging loads (*pln1-4*). Tests of QTL interactions revealed allele-specific epistasis based on high or low strain origin of the QTL alleles. Similar epistatic interactions were found for ovariole number and body size in drosophila (Orgogozo *et al.* 2006; Bergland *et al.* 2008). Again, as with the phenotypic architecture, QTL link the ovaries, VG, sugar perception, and foraging behavior.

The regulation of VG and JH was especially interesting. VG appears to have hormone-like functions by binding to DNA (Gro Amdam and Guyan Harwood personal communication). It suppresses the production of JH and JH in turn suppresses *VG* (Pinto *et al.* 2002; Guidugli *et al.* 2005) establishing a double repressor regulator. JH regulates the onset of foraging behavior and VG affects foraging specialization for pollen and nectar (Page *et al.* 2012). In time, by generation 33, selection in the low strain bees uncoupled the dual feedback inhibition, JH titers were no longer responsive to VG titers. We demonstrated this by knocking down VG in high and low strain workers using dsRNA. High strain bees demonstrated the typical response: elevated JH, earlier onset of foraging, and changes in foraging behavior (Amdam *et al.* 2003, 2007; Ihle *et al.* 2010). As a consequence, the nonresponsive low-strain genotype allowed us to map QTL for the dual suppression regulation to *pln3* (Ihle *et al.* 2015). The gene or genes contained in *pln3* link two physiological modules involved in reproduction, those affecting the production of VG and protein-foraging behavior and those affecting JH and the onset of foraging. Genetic variation for VG/JH regulation has also been found in other populations of honey bees (Antonio *et al.* 2008).

## Positional candidate genes

QTL mapping is an intermediate step to identify regions of the genome that contain genetic factors that influence the traits of interest. Mapped QTL are statistically estimated regions of the genome that contain few-to-many genetic elements, depending on the estimated confidence interval of the size of the region and the distribution of genes in the genome. Once a region is identified, the next step is to compare the region to a genome sequence and try to identify genes contained within the QTL. A goal is to have as few genes as possible. The level of saturation of the map (the number and distribution of markers), the physical size of the genome (number of base pairs), the number of genes in the genome, the number of individuals mapped, and the rate of recombination for the mapped population, all contribute to the number of base pairs and genes found per centimorgan (cM) of genetic map. Crossovers generate the recombinational genotypes needed for mapping. Higher rates of recombination mean fewer genes within a cM of map distance. The honey bee has the highest rate of recombination of any higher eukaryote measured so far. The genetic map is about 4000 cM and the physical size is about 240 Mb, and is estimated to contain about 15,000 genes (Hunt and Page 1995; Beye *et al.* 2006, Weinstock *et al.* 2006; Solignac *et al.* 2007; Elsik *et al.* 2014). That translates into about 60 kb and 3.8 genes per cM. The 97% confidence interval for the four pollen-hoarding QTL spanned about 40 cM and contained 113 predicted genes for the first comparison with the draft sequence (Hunt *et al.* 2007) and contains 219 in OGS3.2.

Examination of the gene lists for each QTL revealed 8 candidate genes that were of special interest based on the known phenotypic architecture (Table 2). They are involved in signaling pathways used in ovary development, VG production, reproductive signaling, sensory response to sugar, and age-based division of labor, key elements in the phenotypic and genetic architectures. Insulin-like signaling (*IIS*) genes were of special interest to us because insulin signaling is necessary for vitellogenesis and is convergent with the ecdysteroid cascade linked to reproduction in drosophila (Richard *et al.* 2005). In addition, it plays a role in insect life span, reproductive status, growth, and metabolism (Tatar *et al.* 2001), and interacts with VG and JH production (Guidugli *et al.* 2005). *IRS* and *PDK1* affect insect endocrine physiology (Flatt *et al.*2005), and in honey bees *IRS* is required for JH synthesis, linking *IIS* to foraging behavior (Wang *et al.* 2010). Our QTL maps showed that *IIS* genes were significantly overabundant within QTL for pollen hoarding (Hunt *et al.* 2007) suggesting that genes work together in the phenotypic architecture of pollen hoarding had, over evolutionary time, aggregated on chromosomes of the honey bee genome (Murren 2012). Similar clustering of insulin-like peptides occurs in drosophila (Tatar *et al.* 2003), however, we have no evidence of synteny.

**Table 2:** Candidate genes for *pln* QTL

QTL	Sequence interval OGS3.2	Physical size of confidence interval^*a*^	Total number of predicted genes	Genes/Mb	Genes of interest
*pln1*	Chr 13 6,602,632–8,363,181	3.4 Mb (1.8 Mb)	18 (70)	5 (20)	Phosphatidylinositolglycan-peptide *(PIG-P), bazooka* (*baz*)
*pln2*	Chr 1 15,641,200–17,973,359	2.1 Mb (2.3 Mb)	59 (91)	28 (40)	1- phosphatidylinositol-4-phosphate 5-kinase (*PIP5K*), Hormone receptor-like in 46 (*Hr46*), tyramine receptor 1 (*TYR1*)
*pln3*	Chr 1 7,074,309–8,363,181	1.4 Mb (1.3 Mb)	32 (53)	23 (41)	Phosphoinositide-3 kinase class I or II (*P13K II*), 3-phosphoinositide-dependent kinase 1 (*PDK1*)
*pln4^b^*	Chr 13 10,002,783–10,187,350	0.1 Mb (0.2 Mb)	4 (5)	40 (25)	*Insulin receptor substrate (IRS)*

a Parentheses denote data from OGS3.2.

b pln4 is located near the end of a linkage group for which we lacked sufficient flanking markers to determine a confidence interval. Also some markers greatly expanded the map resulting in the small estimates of physical distance. Initially we selected a 10 cM region around it from the draft sequence to scan for predicted genes (Hunt *et al.* 2007). We subsequently scanned a similar interval from OGS3.2.

*HR46* was also a gene of interest. Contained in *pln2*, *HR46* is a presumed nuclear hormone receptor that binds ecdysteroids that then enter the nucleus as transcription factors (Simonet *et al.* 2004). It is believed that *HR46* integrates JH and *VG* effects on growth, development, and behavior downstream from insulin signaling. *HR46* may also act via *FTZ1-F1* in apoptotic signaling during larval development and affect ovariole number in adults, a major determinant of VG titers and foraging behavior.

*TYR1*, like *HR46*, is located on chromosome 1 inside of the *pln2* QTL confidence interval. The two genes are tightly linked at a physical distance of 250 kb, or about 5 cM. Recombination events occur only 5% of the time between these genes. Tyramine receptor (TYR1) and its ligand, tyramine (TA) have roles in reproductive physiology, development, and behavior. TA affects the response of bees to sucrose solution (Scheiner *et al.* 2002), motor behavior (Fussnecker *et al.* 2006), learning and memory (Behrends and Scheiner 2012), and ovary physiology (Lehman *et al.* 2006; Salomon *et al.* 2012). Tyramine has also been shown to affect the transition of sterile worker bees into reproductive workers in colonies without queens (Sasaki and Harano 2007). All of these effects are part of the phenotypic architecture of pollen hoarding behavior.

## Verifying candidates

### Expression assays

Candidate genes need to be qualified before investing in further validation. The first step was looking at genes that made sense with respect to what we know, as shown above. Next, we looked for differences in gene expression between workers in the high and low strains. Gene expression comparisons, however, are limited because they do not account for potential functional differences in genes or *cis*- versus *trans*- regulation, but are relatively easy to do. Gene expression assays revealed age and tissue-specific differential expression between high and low strain adult bees for *PDK1* (*pln3*), *HR46* (*pln2*), and *TYR1* (*pln2*). *HR46* was differentially expressed in larvae, in the ovaries and fat body of newly emerged adults, and the fat body of foragers. The ovaries produce ecdysteroids that initiate vitellogenesis in the fat body, linking these two tissues in reproductive signaling (Amdam *et al.* 2010). *PDK1* was differentially expressed in the fat body of foragers while *TRY1* was differentially expressed in the brain, fat body, and ovaries of high and low strain adult bees (Hunt *et al.* 2007; Wang *et al.* 2009, 2012, 2020; Page *et al.* 2012).

A clear link exists between ovary size, *HR46, PDK1*, and foraging behavior. Expression assays of positional candidates were conducted on high-strain backcross bees, (H male) × (HXL queen), with more (large ovary) and fewer (small ovary) ovarioles. High-strain bees have more ovarioles than low-strain bees. Because of male haploidy, the backcross mating scheme dissociated genetic linkages between the genes that affect ovary size, and *HR46* and *PDK1.* After comingling the high- and low-strain genomes, backcross workers with more ovarioles (derived from the high strain) were more likely to collect pollen, forage earlier in life, and collect nectar of higher sugar concentration (Wang *et al.* 2009). Expression differences were found between large and small ovary-sized, young adult bees for *HR46* and for large and small ovary-sized foragers for *PDK1*. Expression dynamics of *HR46* and *PDK1* suggest different roles operating in different stages of adult life. *HR46* is differentially expressed between bees with large and small ovaries soon after they emerge and may be involved in the initiation and production of VG while *PDK1* is differentially expressed in foragers and may be involved in foraging choice decisions. *HR46* and *PDK1* expression is linked to the size of the ovary. Ovary development is completed before bees emerge as adults, therefore, *HR46* and *PDK1* expression in adults are both temporally downstream of ovary development, and their independent candidacy as genes within QTL responsible for the observed foraging differences is unverifiable unless they also affect the size of the ovary during development, or we can effectively knockdown their expression in adults.

### Candidate gene knockdown

How does one make a convincing argument that any positional candidate found inside a mapped QTL was an actual target of selection? Expression assays on adult bees cannot be conclusive because the ovary confounds expression results. The number of ovarioles maps to the same QTL as those affecting the social pollen hoarding phenotype and individual foraging behavior. The ovary could be controlling expression of those genes in adults. We need to be able to knockdown target genes.

Gene knockdown by RNA interference has been used with honey bees since 2003 when it was employed to successfully knock down *VG* in adults (Amdam *et al.* 2003) and *CSD* in larvae (Beye *et al.* 2003). Larval gene knockdown also has been successful for *IRS*, *TOR*, and *USP* (Wang *et al.* 2010; Mutti *et al.* 2011; Ament *et al.* 2012) but is problematic for studying behavior because it cannot, at least so far, be done in the nest but instead requires rearing larvae in the lab on manipulated diets. Lab diets produce workers that are often not normal, frequently having more ovarioles than nest-reared bees (Leimar *et al.* 2012). The use of RNAi is also limited because of the difficulty of getting dsRNA incorporated into target tissues such as the brain and ovary (Page *et al.* 2012), and the effects are ephemeral. However, the fat body, functionally like our liver, is very receptive to incorporating dsRNA and is the target for our knockdowns because it is where VG is produced.

We attempted to knock down *HR46* and *PDK1* in the fat body of adult workers but failed to get a consistent result. However, we were successful with *TYR1* and looked for effects on signaling pathways that may lie between *TYR1* and *VG* (Wang *et al.* 2020). *VG* transcription was significantly reduced, as were genes involved in regulatory pathways (Table 3). One effect of tyramine signaling in the fat body appears to be to upregulate key receptors for ecdysteroid, a primary signal for initiation of vitellogenesis, insulin signaling, and adipokinetic hormone, all involved in VG production and JH signaling and regulation.

**Table 3:** Gene expression in the fat body of adult workers after *TYR1* dsRNA treatment

Related pathway	Gene	Gene name
TA	TYR1*	Tyramine receptor 1
Reproductive pathway	VG*	Vitellogenin
Insulin pathway	*ILP1*	Insulin peptide 1
	*ILP2*	Insulin peptide 2
	INR1*	Insulin receptor 1
	INR2*	Insulin receptor 2
AKH	*AKH*	Adipokinetic hormone
	AKHR*	Adipokinetic hormone receptor
Ecdysone	HR46*	Ecdysone receptor
	FTZ-F1*	Ecdysone receptor
JH	JHE*	JH esterase
	*KR-H1*	Krüppel homolog 1

* Demonstrated a significant decrease in expression with TYR1 knockdown.

One might assume that all of the candidates affect only ovary development and the other traits are consequences of ovary signaling. However, a QTL may contain candidate genes that independently or jointly affect ovary development and behavior. Candidate genes also may have more than one effect, acting in more than one life stage or age. Positional candidates *PDK1*, *HR46*, and *TYR1*, operate in complex signaling networks that are used in many ways during development, metabolism, physiology, and behavior. TYR1 may affect foraging behavior through its effects on VG production in adult workers as demonstrated in the knockdown experiment. We fed tyramine (a ligand for TYR1) to developing larvae during a critical developmental window when ovariole number is determined and increased the number of ovarioles when they emerged as adults. (Wang *et al.* 2020). This demonstrates a role for TA/TYR1 in ovary development, potentially affecting all downstream behavioral traits. Late instar larvae contain ovaries with more than 100 primordial ovarioles. During instar 5 and early pupal development the ovarioles undergo apoptosis in worker larvae, but not queens. The queen ovarioles are rescued from cell death by JH. Queen larvae have elevated titers (Schmidt-Capella and Hartfelder 1998), suggesting a role for TA in JH production in developing larvae. Effects of QTL may be under joint selection in different tissues and life stages.

## Conclusions

Can we get there from here: from variation in complex social behavior to variation in genes and gene networks? Here, I describe a forward genetic approach using human-assisted colony-level selection for a social phenotype, individual behavioral phenotyping, QTL mapping, candidate gene selection, and gene expression and gene knockdown studies. These studies revealed a phenotypic and genetic architecture of a complex social trait. Colony-level selection reverse engineered and integrated the phenotypes of workers for traits that are involved in reproductive and sensory physiology with foraging behavior. Foraging division of labor is at least to some extent determined by reproductive genetic and phenotypic networks of evolutionarily ancient origin, suggesting that natural selection for food storage and foraging division of labor was conservative.

QTL mapping produced genotype–phenotype maps rich in pleiotropy, epistasis, and epistatic pleiotropy revealing a complex genetic architecture matching the architecture of individual phenotypes, suggesting that phenotypic integration is supported by pleiotropy or tight linkage within mapped QTL. Examination of the degree of genetic determination of individual and colony-level phenotypes demonstrated that the social and anatomical phenotypes had similar levels of genetic determination, about 40%, while individual behavioral traits were about 5–16%. The social pollen hoarding trait is closely regulated by colonies of bees as is the anatomical trait, size of ovary, during development. The individual behavioral traits are subject to much more variance, thereby, reducing the amount of variance explained by their genotypes.

Gene expression, gene association, and knockdown studies revealed 3 positional candidate genes of special interest, *TYR1* and *HR46* (*pln2*), and *PDK1* (*pln3*). Effects of *HR46* and *PDK1* have been demonstrated in adult bees but have not yet been tested in developing larvae. All QTL demonstrate effects on ovary size and ovary size affects all measured foraging behavior traits. Ovary size is determined in the larval stage. Therefore, it is necessary to test candidates in both life stages. TYR1 has been shown in knockdown and pharmacological studies to affect transcription of *VG* and the size of the ovaries, verifying it as a pleiotropic gene within *pln2* that was selected in two different tissues (ovary and fat body) in two different life stages (larva and adult) during colony-level selection for pollen hoarding.

This was a 30-year effort limited ultimately by our ability to study the effects of individual genes. Tissue and developmental stage-specific targeted knockdowns are critical to construct gene networks with directional paths of causality. Sufficient statistical methods for analyzing causal paths of gene action are likewise needed (Shipley 2000). Targeted gene knockout and knockdown in honey bees has been elusive. Methods that have been successful in other organisms, such as the use of transposable elements, have not worked, although limited success has been demonstrated with *piggyBac*-derived cassettes (Schulte *et al.* 2014; Otte *et al.* 2018). CRISPR/Cas9 has recently been used to edit targeted genes (Kohno *et al.* 2016; Roth *et al.* 2019; Değirmenci *et al.* 2020). To be useful for studying behavior, *piggyBac* and CRISPR/Cas9 require injection of eggs containing embryos that will be raised as transformed queens that produce transformed worker and drone offspring. The effects of the transformation should be tissue and developmental-stage specific. Transformation success is highly variable, a difficulty when a high percentage of transformed workers is necessary for studies of social behavior. It is difficult to raise transformed bees within the nest because adult workers easily detect any abnormalities of larvae and adults and eliminate them. *In vitro* rearing is a problem because rearing environment is an important determinant of adult anatomy and consequently behavior (Leimar *et al.* 2012). Though not yet a “go to” technique, CRISPR/Cas9 techniques continue to evolve and improve and look promising as future tools for both forward and reverse genetic analyses.

## Conflicts of interest

None declared.
